# RFG-TVIU: robust factor graph for tightly coupled vision/IMU/UWB integration

**DOI:** 10.3389/fnbot.2024.1343644

**Published:** 2024-04-29

**Authors:** Gongjun Fan, Qing Wang, Gaochao Yang, Pengfei Liu

**Affiliations:** ^1^CCCC Investment Company Limited, Beijing, China; ^2^School of Instrument Science and Engineering, Southeast University, Nanjing, China; ^3^School of Computer and Artificial Intelligence, Changzhou University, Changzhou, China

**Keywords:** vision/IMU/UWB, factor graph, robust, adaptive estimation, pre-calibration

## Abstract

High precision navigation and positioning technology, as a fundamental function, is gradually occupying an indispensable position in the various fields. However, a single sensor cannot meet the navigation requirements in different scenarios. This paper proposes a “plug and play” Vision/IMU/UWB multi-sensor tightly-coupled system based on factor graph. The difference from traditional UWB-based tightly-coupled models is that the Vision/IMU/UWB tightly-coupled model in this study uses UWB base station coordinates as parameters for real-time estimation without pre-calibrating UWB base stations. Aiming at the dynamic change of sensor availability in multi-sensor integrated navigation system and the serious problem of traditional factor graph in the weight distribution of observation information, this study proposes an adaptive robust factor graph model. Based on redundant measurement information, we propose a novel adaptive estimation model for UWB ranging covariance, which does not rely on prior information of the system and can adaptively estimate real-time covariance changes of UWB ranging. The algorithm proposed in this study was extensively tested in real-world scenarios, and the results show that the proposed system is superior to the most advanced combination method in all cases. Compared with the visual-inertial odometer based on the factor graph (FG-VIO), the RMSE is improved by 62.83 and 64.26% in scene 1 and 82.15, 70.32, and 75.29% in scene 2 (non-line-of-sight environment).

## 1 Introduction

With the advent of the information age, localization-based services (LBS) have played an increasingly important role in various application scenarios (Suhr et al., 2017). In outdoor scenes, the global navigation satellite system (GNSS) can provide reliable and stable global positioning and navigation services. However, satellite signals are lost in many indoor scenes, such as underground garages, traffic tunnels and urban canyons, which have spawned many indoor positioning technologies (Schreiber et al., 2016; Gao et al., 2017). Visual simultaneous localization and mapping (VSLAM) use the visual system to extract different images in the process of camera movement by detecting the changes in these different images, extracting and matching the same feature points, and judging the motion changes of the feature points to estimate the motion of the camera (Zhang et al., 2014). Because visual odometry (VO) cannot track well in the face of simple rotation, it is usually combined with low-cost inertial sensors such as inertial measurement units (IMU) in practical applications. Visual inertial odometry (VIO) can be robustly used after combination (Usenko et al., 2016; Xu et al., 2021; Yang et al., 2021).

Because of the lack of a global position information reference, although VIO has high positioning accuracy under good lighting conditions and good image quality, there is a problem of cumulative error (Cheng et al., 2014). Therefore, many studies have been conducted to improve the applicability of visual localization in long-distance ranges by designing global landmarks or supplementing them with other global information. Sensors that can perceive global information, such as GNSS, magnetometers and Ultra-wide band (UWB), are global sensors. Theoretically, combining a VIO with high local accuracy but accumulated errors and a global sensor with guaranteed local accuracy without accumulated errors can compensate for each other’s shortcomings (Paul and Kyle, 2018). Mascaro et al. proposed a multi-sensor loosely coupled method based on filtering (Mascaro et al., 2018). The main idea is to use the IMU as the main sensor and obtain a pose with 6-degrees of freedom (DOF) by integration. VO/VIO is used as the relative pose estimator, GNSS as the global pose estimator and an extended Kalman filter (EKF) is performed with the result obtained by IMU integration to obtain a more accurate position estimation. Moreover, GNSS does not have the problem of a cumulative error, which can correct the cumulative error of the IMU and VO/VIO. Li et al. added GNSS/IMU information to the VSLAM framework, constructed a propagation equation for the GNSS and IMU data between two frames of images and finally solved the optimal estimation through graph optimization (Li et al., 2019). Patrick et al. processed the vision and laser point cloud information through the ORB-SLAM2 and LOAM algorithms, added them to the fusion framework and then fused them with GNSS and prior maps to obtain real-time positioning and mapping (Patrick et al., 2018). For different application platforms, Zheng et al. applied factor graph fusion to Unmanned Aerial Vehicle (UAV) positioning to realize the fusion of IMU, GNSS, barometers and optical flow (Zheng et al., 2016). Due to inability to receive GNSS signals indoors, the accumulated error of the VIO cannot be eliminated by GNSS indoors. UWB indoor positioning technology based on ranging information is widely used and has high resolution and accuracy. Therefore, indoors, UWB can be used to eliminate the error accumulation of the camera/IMU. However, owing to factors such as the performance of UWB electronic devices, indoor multipath and non-line-of-sight (NLOS) propagation, UWB ranging contains errors that affect the positioning accuracy (Song et al., 2019).

Most traditional fusion methods use KF (Mourikis, 2007; Chen et al., 2018). However, the filtering method discards historical information. The factor graph optimization method optimizes the current and historical information by constructing a factor graph. Through repeated iterations, factor graph optimization can reduce linearization errors and approach the optimal solution better. Because nonlinear optimization can simultaneously optimize the data of multiple time periods, it is better than the algorithm based on filtering (Du, 2012; Bresson et al., 2016). This study focuses on a fusion algorithm based on factor graph optimization. The factor graph model has strong flexibility and can realize the “plug-and-play” of sensors, which has received extensive attention in the field of navigation (Guowei et al., 2018). Indelman et al. (2013) realized the information fusion of IMU, GNSS and vision based on factor graphs and incremental smoothing. Based on a monocular camera, IMU and Lidar, Shao et al. combined tightly coupled VIO and Lidar mapping modules and used factor graph optimization to obtain a real-time 6-DOF pose estimation (Shao et al., 2019). Mikhail et al. employed infrared cameras, binocular cameras with LED lights, IMUs and lidar sensors to handle localization in visually degraded environments in a factor-graph framework (Mikhail et al., 2019). Nguyen proposed a tightly coupled scene with a monocular camera, a 6-DoF IMU and a single unknown UWB anchor to achieve accurate and drift-reduced localization (Li and Wang, 2021; Nguyen et al., 2021). Hu et al. proposed a tightly-coupled fusion of a monocular camera, a 6-DoF IMU and multiple position-unknown UWB anchors to construct an indoor localization system (Hu et al., 2023). When UWB ranging anomalies are detected, the system will dynamically discard these observations. Liu et al. proposed a tightly coupled integration algorithm of GNSS RTK, UWB and VIO to enhance the accuracy and reliability for UAV seamless localization in challenging environments (Liu et al., n.d.). Kao et al. proposed a learning-based UAV localization method using the fusion of vision, IMU, and UWB sensors, which consisted of VIO and UWB branches. The model combined the estimation results of both branches to predict global poses (Kao et al., 2023). Similar methods can also be found in (Dong et al., 2022; Ochoa-de-Eribe-Landaberea et al., 2022).

The most significant deficiency in the factor graphs is the distribution of weights. In the algorithm, the noise variance matrix of the initial measurement information of each sensor is obtained according to experience and different weights are assigned to the corresponding observations. However, in an actual system, there is uncertainty in the observation information; that is, the variance of the observation noise changes. Therefore, the weight assignment method relies excessively on the initial experience value and the weight value will not change dynamically with the actual situation (Wei et al., 2021). In general, if the accuracy of a specific sensor is higher, a larger weight is assigned based on experience. In the data fusion process, even if its performance suddenly deteriorates, its observational information weights will not change, leading to poor results. Based on the above analysis, we can draw the following conclusion that the traditional factor graph has serious problems in the distribution of the weights of the observation information. Therefore, this study improves the algorithm of the traditional factor graph fusion method.

To address these problems, taking the robot indoor navigation and positioning system as the research object and focusing on the information fusion technology of the integrated navigation system, this paper proposes a factor graph fusion algorithm with dual functions of weight adjustment and gross error elimination to realize a tight combination of camera, IMU and UWB. Changing the size of each sensor’s observation noise covariance matrix suppress the influence of observation anomalies and the positioning accuracy and robustness of the integrated navigation are improved. The contributions of this study are as follows:

➢ To overcome the defects of single-sensor positioning and achieve robust indoor positioning, we propose a robust factor graph model to realize the tight combination of Vision/IMU/UWB (VIU), which is called RFG-TVIU and give the corresponding Jacobian matrix derivation. At the same time, to solve the long-term NLOS effect of UWB, according to the NLOS error characteristics, we propose adding UWB’s adjacent time differential ranging to the back-end constraints of the system and designing a smoothing method to remove UWB noise.
➢ Based on redundant measurement information, we propose a novel adaptive estimation model for UWB ranging covariance, which does not rely on prior information of the system and can adaptively estimate real-time covariance changes of UWB ranging; Meanwhile, to solve the weight allocation problem in multi-sensor fusion, a robust factor graph model is proposed.
➢ Finally, we compare RFG-TVIU with three other models, including FG-VIO (VINS-Mono without loop) (Qin Tong et al., 2017), IMU/UWB (UWB hardware’s own IMU/UWB fusion algorithm, a relatively stable UWB localization algorithm) and FG-TVIU (VIU with tightly coupled based on factor graph) (Xie et al., 2022) through several different scenes and present the comparative analysis results.

The structure of this paper is organized as follows: The first part is the introduction and the second part will first introduce the tightly coupled VIU based on a factor graph and give the corresponding mathematical derivation. The third part covers the development of a robust factor graph model based on the sliding window online estimation of factor graph weights. The fourth part includes experimental verification and result analysis and the fifth part draws a conclusion and proposes prospects.

## 2 A new factor graph model for tightly-coupled VIU

The graph optimization algorithm can obtain a smooth travel trajectory for the carrier during the entire operation process. The navigation information at all time points was estimated and optimized several times and the result was highly accurate. Therefore, the navigation and positioning algorithm based on a factor graph is used in the real-time positioning and composition of vision and lidar sensors have been widely used. This section describes the factor graph algorithm framework and the VIU multi-sensor fusion algorithm. Factor graph models of various vehicle navigation sensors were constructed based on the analysis of the performance of various navigation sensors. The navigation sensors used in this study mainly include an IMU, camera and UWB.

### 2.1 Overview of the tightly coupled monocular VIU system

**Figure 1:**
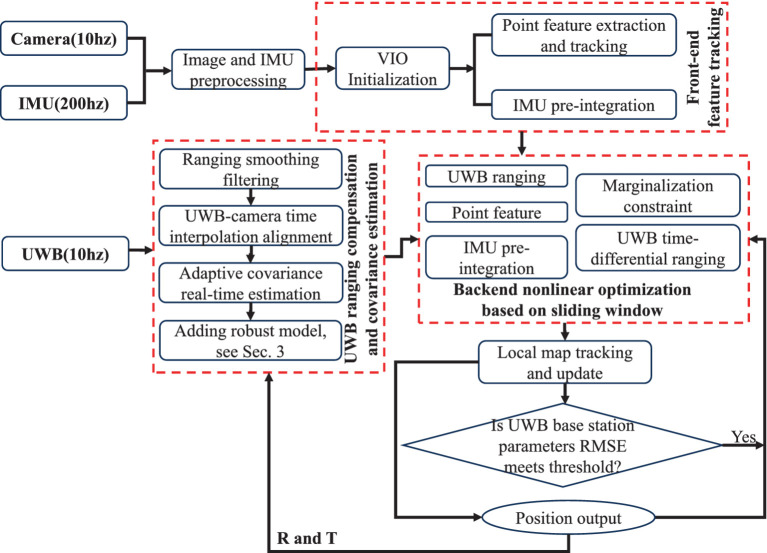
Overview of the tightly coupled monocular VIU system.

According to the description above, an overview of the proposed VIU with tightly coupled is shown in Figure 1. After initialization of the integrated system Vision/IMU, the INS mechanism begins to provide a high-rate navigation output, including the position, velocity and attitude. The features on the plane are mapped to the 3-Dimensions (3D) space, and the 3D structure of the scene is restored using structure from motion (SFM); then, tracking and pose calculations are performed according to the established map. Before discussing the measurement models and estimation algorithms for the bundle adjustment (BA) and navigation filter, it is appropriate to first introduce the state vectors for each estimator (Yang and Shen, 2017).

The camera collects the state vector, and the optimization equation increase with time and the images. If the system runs for a long time, it is easy to encounter the problem of dimensional disasters, which makes the system unable to process the data in real time. Therefore, an optimization method based on a sliding window is generally designed, and the number of optimized frames is fixed using a sliding window to limit the computational complexity of the system. In addition, the marginalization method was designed for the state quantity of the sliding window, and its constraints were retained. In this study, we set the size of the sliding window to N (N = 10), assuming that χ represents the state parameters that must be estimated at time i.


(1) $$\{\begin{array}{c} \chi =\left[\begin{array}{c} x_{i},x_{i+1},\cdots ,x_{i+N},\lambda_{a},\lambda_{a+1},\cdots ,\lambda_{a+A},\delta T_{d},\\ p_{b}^{c},q_{b}^{c},p_{u,}^{b}P_{1,}^{w}P_{2,}^{w}\cdots ,P_{m}^{w} \end{array}\right]\\ x_{i}=\left[p_{b_{i}}^{w},q_{b_{i}}^{w},v_{b_{i}}^{w},b_{a}^{b_{i}},b_{g}^{b_{i}}\right] \end{array}$$

where b is the body coordinate frame, which is consistent with the IMU coordinate system; w is the world coordinate frame; $p_{b_{i}}^{w}$ , $q_{b_{i}}^{w}$ and $v_{b_{i}}^{w}$ are the position, speed and rotation of the ith IMU state in the world frame, respectively. λ is the inverse depth of the feature points to be estimated. $p_{b}^{c}$ and $q_{b}^{c}$ are the translation and rotation matrices from the camera to the IMU, respectively. $p_{u}^{b}$ is a translation matrix from UWB tag to IMU. The subscripts i and a are the start indices of the IMU states and point landmarks, respectively. A is the number of point landmarks observed by all keyframes in the sliding window. ${\delta T}_{d}$ is the time-equivalent error delay between the UWB and the IMU. $P_{m}^{w}$ is the coordinates of the UWB base station to be estimated. The subscript m represents the number of base stations. To improve the efficiency of system nonlinear optimization, when the RMSE of UWB base station coordinates converges to a certain threshold, the base station coordinates are kept fixed during back-end optimization.

According to the factor graph definition, we can construct the measurement residuals model with the pose of the current frame that needs to be optimized and solve it by minimizing the following cost function:


(2) $$\begin{array}{l} min\;\rho \left({\left\|r_{p}-J_{p}\chi \right\|}_{\Sigma_{p}}^{2}\right)+\sum_{i\in B}\rho \left({\left\|r_{b}\left(z_{b_{i}}^{b_{i+1}},\;\;\chi \right)\right\|}_{\Sigma_{b_{i}}^{b_{i+1}}}^{2}\right)\\ +\sum_{\left(i,j\right)ÎF}\rho \left({\left\|r_{f}\left(z_{f_{j}}^{c_{i}},\;\;\chi \right)\right\|}_{\Sigma_{f_{j}}^{c_{i}}}^{2}\right)+\sum_{\left(i,j\right)ÎU}\rho \left({\left\|W\left(s_{U_{j}}\right)r_{U}\left(z_{U_{j}}^{c_{i}},\chi \right)\right\|}_{\Sigma_{U}}^{2}\right) \end{array}$$

where Σ is the covariance factor, which is related to the weighting factor of this measurement; $\left\{r_{p},J_{P}\right\}$ is prior information that can be computed after marginalizing a frame in the sliding window, and $J_{P}$ is the prior Jacobian matrix from the resulting Hessian matrix after the previous optimisation. $r_{b}\left(z_{b_{i}}^{b_{i+1}},\chi \right)$ is an IMU measurement residual between body state $x_{i}$ and $x_{i+1}$ . $r_{f}\left(z_{f_{j}}^{c_{i}},\chi \right)$ is a point feature reprojection residual. $r_{s}\left(z_{s_{j}}^{c_{i}},\chi \right)$ includes UWB ranging measurements and time-differential ranging measurement residuals.

The vehicle navigation sensors used in this study include the IMU, monocular camera and UWB wireless positioning system. In this section, the sensor information is abstracted into measurement factors based on the factor graph theory. The schematic of the localization of the combined VIU system is shown in Appendix Figure A1. The yellow polygonal represents the pre-integration information of the IMU. The dark green rectangles represent the base stations of the UWB sensors, which are able to communicate with the sensor tags carried on the carrier and calculate the distance between the base station and the tags. The blue dotted line indicates that NLOS ranging observations. The red dotted line indicates that LOS ranging observations. The blue pentagons represent the point-feature constraints the combined camera system provides between the two moments.

The measurement error models of the feature points, IMU pre-integration and UWB ranging are introduced below. First, error models of the feature points factor node are given.

### 2.2 Point feature measurement model of camera

The vision observation residual is the reprojection error of the camera feature points, which lies between the estimated value of the projected position and the observed value in the normalized camera coordinate system. First, the i_th_ frame where the first landmark point P is observed is converted to the pixel coordinates observed by the corresponding landmark point P in the j_th_ frame, and the vision observation residual is established as follows:


(3) $$r_{f}\left(z_{f_{k}}^{c_{j}},\chi \right)={\left[b_{1}\;\;b_{2}\right]}^{T}\left({\bar{P}}_{f_{k}}^{c_{j}}-P_{f_{k}}^{c_{j}}/P_{f_{k}}^{c_{j}}\right)$$

(4) $${\bar{P}}_{f_{k}}^{c_{j}}=\kappa_{c}^{-1}{\left[\begin{array}{cc} u_{f_{k}}^{c_{j}} & v_{f_{k}}^{c_{j}} \end{array}\right]}^{T}$$

(5) $$P_{f_{k}}^{c_{j}}=R_{b}^{c}\left(R_{w}^{b_{j}}\left(R_{b_{i}}^{w}\left(R_{c}^{b}1/\lambda_{k}\kappa_{c}^{-1}{\left[\begin{array}{cc} u_{f_{k}}^{c_{i}} & v_{f_{k}}^{c_{i}} \end{array}\right]}^{T}+P_{c}^{b}\right)+P_{b_{i}}^{w}-P_{b_{j}}^{w}\right)-P_{c}^{b}\right)$$

Given that the point feature needs to use the state and pose parameters at two times when projecting, the measurement information establishes a relationship with the two-factor nodes at the current moment and the previous moment, and its factor graph model is shown in Appendix Figure A2.

### 2.3 Measurement model based on pre-integration of IMU

As the primary navigation device of the current navigation system, the IMU has a high information-update frequency. If a factor node is established for each inertial navigation information, the amount of calculation is large and time-consuming. Therefore, at present, for establishing the inertial navigation system factor graph, only the navigation state quantity that needs to be output for the measurement is set as a variable node, and the IMU factor nodes at two adjacent moments are redefined. The pre-integration algorithm integrates the obtained carrier motion state information under the machine system, which can effectively improve the real-time performance of the algorithm (Chang et al., 2020).

The IMU generated the observation residual of the IMU between consecutive frames in the sliding window. Considering the IMU measurement between two consecutive frames, a and b, as shown in Eq. (6), the residual variable that must be optimized is the position α between the two frames, β , θ and IMU bias $b_{a}$ and $b_{g}$ .


(6) $$r_{b}\left({\hat{z}}_{b_{j}}^{b_{i}},\chi \right)=\left[\begin{array}{c} \delta \alpha_{b_{j}}^{b_{i}}\\ \delta \theta_{b_{j}}^{b_{i}}\\ \delta \beta_{b_{j}}^{b_{i}}\\ \delta b_{a}\\ \delta b_{g} \end{array}\right]={\left[\begin{array}{c} R_{w}^{b_{i}}\left(p_{b_{j}}^{w}-p_{b_{i}}^{w}-v_{i}^{w}\Delta t+\frac{1}{2g^{w}\Delta t^{2}}\right)-{\hat{\alpha }}_{b_{j}}^{b_{i}}\\ 2{\left[{\left({\hat{q}}_{b_{j}}^{b_{i}}\right)}^{-1}⊗{\left(q_{b_{i}}^{w}\right)}^{-1}⊗q_{b_{j}}^{w}\right]}_{xyz}\\ R_{w}^{b_{i}}\left(v_{j}^{w}-v_{i}^{w}\Delta t+g^{w}\Delta t-{\hat{\beta }}_{b_{j}}^{b_{i}}\right)\\ b_{a}^{b_{j}}-b_{a}^{b_{i}}\\ b_{g}^{b_{j}}-b_{g}^{b_{i}} \end{array}\right]}_{15\times 1}$$

The Eq. (6) is the pre-integration of the IMU measurements. This is only related to the deviation of the IMU, cutting off the connection with the position, speed and direction of the previous moment. Unless the bias has shifted significantly, it is entirely possible to adjust it using a first-order approximation of the pre-integration term for Eq. (7) below. This has the benefit of reducing the number of computations with little impact on accuracy.


(7) $$\{\begin{array}{c} \alpha_{b_{j}}^{b_{i}}={\hat{\alpha }}_{b_{j}}^{b_{i}}+J_{\delta b_{a}^{b_{i}}}^{\delta \alpha_{b_{j}}^{b_{i}}}\delta b_{a}^{b_{i}}+J_{\delta b_{g}^{b_{i}}}^{\delta \alpha_{b_{j}}^{b_{i}}}\delta b_{g}^{b_{i}}\\ \beta_{b_{j}}^{b_{i}}={\hat{\beta }}_{b_{j}}^{b_{i}}+J_{\delta b_{a}^{b_{i}}}^{\delta \beta_{b_{j}}^{b_{i}}}\delta b_{a}^{b_{i}}+J_{\delta b_{g}^{b_{i}}}^{\delta \beta_{b_{j}}^{b_{i}}}\delta b_{g}^{b_{i}}\\ q_{b_{j}}^{b_{i}}={\hat{q}}_{b_{j}}^{b_{i}}⊗{\left[\begin{array}{cc} 1 & \frac{1}{2J_{\delta b_{g}^{b_{i}}}^{\delta \theta_{b_{j}}^{b_{i}}}\delta b_{g}^{b_{i}}} \end{array}\right]}^{T} \end{array}$$

Eq. (7) is the discrete form of the state equation of the IMU system, which can be expressed as:


(8) $${\hat{\chi }}_{j}=h\left(\chi_{i},z_{i}\right)$$

**Figure 2:**
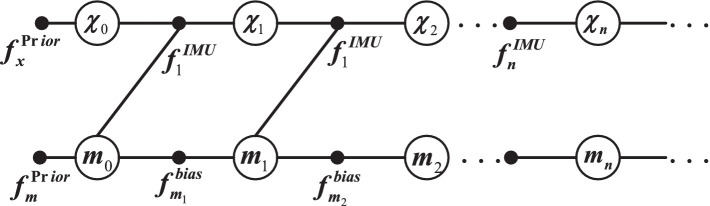
Pre-integration constraint factor graph construction.

When a new measurement node is added to the factor graph, the difference between the estimated and measured values is the cost function that needs to be minimized, and the factor node is established to obtain the expression form of the pre-integration factor node as follows:


(9) $$f^{IMU}\left(\chi_{i},\chi_{j}\right)=d\left(\chi_{j}-h\left(\chi_{i},z_{i}\right)\right),$$

where d(⋅) is the given cost function, $\chi_{i}=\left(x_{i},m_{i}\right)$ and $x_{i}$ and $m_{i}$ are the navigation state and inertial error parameters, respectively.

where $f_{x}^{\mathrm{Pr}ior}$ and $f_{m}^{\mathrm{Pr}ior}$ are the factor nodes formed by the prior information of the navigation state quantity and bias variable of the inertial device in Figure 2, respectively.

### 2.4 Constraints of UWB original and differential ranging observations with time delay online correction

#### 2.4.1 NLOS recognition and compensation based on robust KF

In an indoor environment, UWB signals are refracted and reflected owing to the existence of walls and obstacles, thus increasing the signal transmission time and reducing the positioning accuracy; this is called NLOS. To reduce the noise of UWB ranging and the influence of NLOS measurements, in practical applications, the KF is used to smooth the original range. Taking the distance from the UWB tag to the base station and the speed as state parameters $X_{m,k}={\left[d_{m,k},v_{m,k}\right]}^{T}$ , the equation of state can be obtained as:


(10) $$X_{m,k}=F_{m,k}{\hat{X}}_{m,k-1}+\omega_{m,k},$$

where $F_{m,k}=\left[\begin{array}{cc} 1 & \Delta T_{m,k}\\ 0 & 1 \end{array}\right]$ , $\Delta T_{m,k}$ is the sampling interval of the UWB, $\omega_{m,k}$ is the state noise, and its covariance matrix is $Q_{m,k}$ .

The measurement equation is:


(11) $$Z_{m,k}=H_{m,k}X_{m,k}+\psi_{m,k},$$

where $Z_{m,k}$ is the measurement distance of the base station id m at time k $H_{m,k}=\left[\begin{array}{cc} 1 & 0 \end{array}\right]$ ; $\omega_{m,k}$ is the measurement noise, and its covariance matrix is $R_{m,k}$ .

In the KF, the estimated solution of the innovation vector is $r_{m,k}=Z_{m,k}-H_{m,k}{\dot{X}}_{m,k}$ , and the covariance is $N_{m,k}=H_{m,k}{\dot{P}}_{m,k}H_{m,k}^{T}$ . If there is no NLOS between the UWB base station and tag, it can be considered that $r_{m,k}$ obeys a Gaussian distribution with zero mean; if there is NLOS between the UWB base station and tag, which reduces the ranging accuracy, it can be considered that $r_{m,k}$ satisfies the mean value ${\hat{r}}_{m,k}$ of Gaussian distribution, to construct the verification information as $\sigma_{m,k}=r_{m,k}^{T}P_{m,k}^{-1}r_{m,k}$ , when


(12) $$\{\begin{array}{l} \sigma_{m,k}<c,\;LOS\\ c\leq \sigma_{m,k}<10c\\ \sigma_{m,k}>10c,\;NLOS \end{array},$$

For $c\leq \sigma_{m,k}<10c$ , we use the robust filtering algorithm to reduce the weight of the distance measurement value; c is the empirical value obtained through multiple tests.

#### 2.4.2 Factor graph construction based on UWB constraints

When VIU is tightly coupled, the time deviation between UWB and camera mainly includes two parts: the time stamp misalignment between UWB and Camera and the time delay between UWB and camera. The time stamp misalignment between UWB and camera can be resolved through time interpolation, and the time delay between UWB and camera generally needs to be estimated in real-time as a parameter. Usually, these two types of time errors are coupled together, which make it difficult to decouple them. In response to this situation, this paper adopts a “pseudo-optical flow” method to track UWB ranging. Due to the different $v_{m,k}$ of each UWB base station, the time delay between UWB and camera can be decoupled through this method. Below, we will first introduce the correction of timestamp misalignment between UWB and camera.

Suppose UWB data input is detected at time $t_{1}$ and $t_{2}$ , $b_{i}$ is the camera frame closest to time $t_{1}$ and $t_{2}$ , and $t_{1}\leq b_{i}\leq t_{2}$ . Traditionally, the UWB is used to align the visual observation moment to compensate for the ranging value $d_{m,t_{b_{i}}}$ , as follows:


(13) $$\{\begin{array}{l} w_{1}=\left(t_{b_{i}}-t_{2}\right)/\left(d_{1}+d_{2}\right)\\ w_{2}=\left(t_{1}-t_{b_{i}}\right)/\left(d_{1}+d_{2}\right) \end{array},$$

(14) $$d_{m,t_{b_{i}}}=w_{1}d_{m,t_{1}}+w_{2}d_{m,t_{2}},$$

However, if the UWB frequency is not high or the device is turning sharply, using the interpolation mode to compensate is usually prone to large errors. Therefore, this study adopts the method of aligning the visual observation time with the UWB. We can obtain the pose of the body at time t of UWB sampling as:


(15) $$\{\begin{array}{l} {\hat{p}}_{b,t}^{w}=v_{b_{i}}^{w}\left(t_{b_{i}}-t\right)+p_{b_{i}}^{w}\\ {\hat{q}}_{b,t}^{w}={\left[w_{b_{i}}\left(t_{b_{i}}-t\right)\right]}^{\times }q_{b_{i}}^{w} \end{array},$$

where $v_{b_{i}}^{w}$ is the velocity of the body at time $t_{b_{i}}$ and $w_{b_{i}}$ is the corrected angular velocity at time $t_{b_{i}}$ .

##### 2.4.2.1 Construction of UWB ranging factor graph based on online correction of time delay

UWB generally uses the time-of-flight (TOF) mode for ranging. The UWB, camera and IMU can only use soft synchronization at the front-end; therefore, we need to consider the time delay among them. In this experiment, we used Mynteye’s mono-inertial camera, and synchronization between the camera and the IMU was performed. For more details, please refer to Qin Tong et al. (2017). This study focuses on the online time delay estimation of UWB and camera.

**Figure 3:**
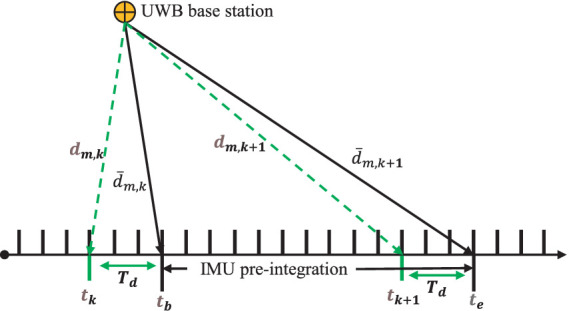
Online estimation of UWB and IMU time delays.

If the time deviation between camera and IMU sensors is not considered, fusing the measurement information obtained at different time will bring errors to the optimization results. The traditional method is to use the delay $T_{d}$ as an amplification state variable for parameter estimation and compensate for the delay error from the output. However, throughout the optimization process, the delay difference during measurement always exists and generates interference, which affects the estimation speed and accuracy of the delay. On this basis, a method is proposed to directly compensate for the delay error of UWB measurement values, introducing the state variable ${\delta T}_{d}$ . The time interval between two reference frames is generally between 0.05 s and 0.1 s. Assuming the distance d of UWB base station $d_{m,k}$ varies uniformly between two reference frames, as shown in Figure 3.

where $t_{k}\;$ and $t_{k+1}\;\;$ are the UWB sampling time; $t_{b}$ and $t_{e}$ are the IMU pre-integration time; $T_{d}$ is the time delay between UWB and camera at time $t_{k}$ ; $d_{m,k}$ and $d_{m,k+1}$ are the original ranging of UWB; ${\bar{d}}_{m,k}$ and ${\bar{d}}_{m,k+1}$ are the ranging after UWB compensation.


(16) $$\delta z_{m,t_{k}}={\bar{d}}_{m,k}-r_{m,t_{k}}+n_{m,t_{k}},$$

(17) $$r_{m,t_{k}}={\hat{p}}_{b_{i}}^{w}+{\hat{q}}_{b_{i}}^{w}t_{u}^{b}-P_{m}^{w},$$

where, $P_{m}^{w}={\left(x_{m}^{w},y_{m}^{w},z_{m}^{w}\right)}^{T}$ is the coordinate parameter of the base station m; ${\bar{d}}_{m,k}$ is the distance measurement of $d_{m,k}$ after time delay compensation; $t_{b_{i}}$ is the timestamp of frame $b_{i}$ ; $t_{u}^{b}$ is the translation matrix from the UWB tag to the IMU body; $p_{b_{i}}^{w}$ is the translation vector of the body at the time i relative to the world coordinate frame (W); $q_{b_{i}}^{w}$ is the rotation vector the body relative to the world coordinate frame at the time i; ∗.norm( ) represents the modulus of the vector.

Before each optimization, compensate for the delay error in UWB measurement and obtain a linear expression about $T_{d}$ . The initial estimated delay $T_{d}$ is set to 0, and an iterative update is performed before each optimization. ${\bar{d}}_{m,t}$ can be obtained as:


(18) $${\bar{d}}_{m,k}=d_{m,k}+v_{m,k}T_{d},$$

where, $v_{m,k}$ is the velocity vector of UWB tag relative to the base station m at time $t_{k}$ .

##### 2.4.2.2 UWB differential ranging constraints at adjacent moments

The position of the UWB tag at any time is constrained only by the measurement distance between the tag and tag. If the measurement is inaccurate, for example, when there is a serious NLOS influence, it is easy to decrease the positioning accuracy. Generally, the output frequency of UWB base stations can reach at least 10 Hz or even greater. By experiments, the NLOS error can be regarded as a constant value in a short time, and the difference in distance between adjacent moments can eliminate the influence of the NLOS error. Therefore, this study uses the distance difference between adjacent moments of the tag as a weak constraint to increase the constraint strength of the tag position, as shown in is shown in Appendix Figure A3. The specific optimization equation is as follows:


(19) $$r_{U}\left(d_{m,k},d_{m,k+1},\chi \right)={\left|\delta {\hat{d}}_{m,k}-\delta {\bar{d}}_{m,k}\right|}_{\overset{k+1}{\underset{k}{\sum }}}^{2},$$

where $\delta {\hat{d}}_{m,k}={\hat{d}}_{m,k+1}-{\hat{d}}_{m,k}$ , $\overset{k+1}{\underset{k}{\sum }}=2\sigma_{m,k}^{2}$ ; ${\hat{d}}_{m,k+1}$ and ${\hat{d}}_{m,k}$ are the distances inversely calculated by the known tag coordinates and the UWB base station coordinates at time $t_{k}$ and $t_{k+1}$ , respectively. Nodes (1) and (2) can be written uniformly as


(20) $$\delta r_{m,k}^{U}=h^{U}\left(\chi_{k},\delta T_{d}\right)+n^{U},$$

**Figure 4:**
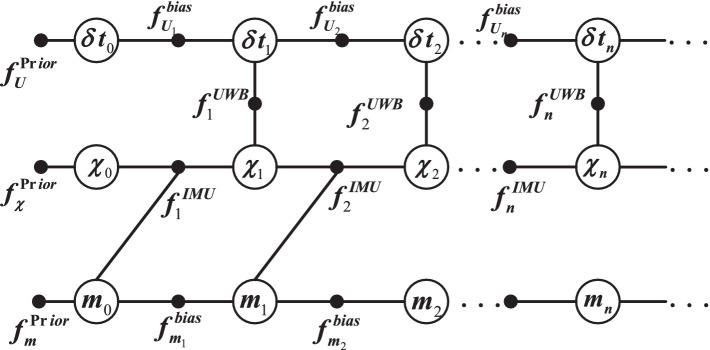
UWB ranging and inter-frame difference constraint factor graph construction.

When a new measurement node is added to the factor graph, establishing a different relationship between the estimated and measured values is the current cost function that must be minimized. To establish factor nodes, we can obtain the expression form of the UWB ranging and UWB differential ranging factor nodes as follows:


(21) $$f^{U}\left(\chi_{k},\delta T_{d}\right)=d\left(z_{m,t_{k}}^{U}-h^{U}\left(\chi_{k},\delta T_{d}\right)\right),$$

where $T_{d}$ is the time delay between UWB and IMU; $n^{U}$ is ranging noise; $f^{U}\left(\chi_{k},\delta T_{d}\right)$ connects the state variable node $\chi_{k}$ and the error variable node $\delta T_{d}$ at the time $t_{k}$ ; $f^{U\_bias}$ is the ranging compensation based on time delay $T_{d}$ at time $t_{k}$ . The factor graph model is shown in Figure 4.

#### 2.4.3 Feedback and compensation of UWB raw ranging

After the factor graph is optimized, it is determined using the residual information. Assuming that base station m has an NLOS error, we use the optimized UWB tag position and the position of the base station m to inversely calculate the distance ${\hat{d}}_{m,k}^{w}$ between the tag and base station


(22) $$\left|p_{b_{i}}^{w}+q_{b_{i}}^{w}t_{u}^{b}+v_{b_{i}}^{w}dt-P_{m}^{w}-d_{m,k}^{w}\right|\geq s\times v_{b_{i}}^{w}\Delta t,$$

If this inequality is established, the measured value $d_{m,k}^{w}$ is abnormal. At the same time, the threshold can be adjusted by the node factor s. ${\hat{d}}_{m,k}^{w}$ can be obtained as:


(23) $${\hat{d}}_{m,k}^{w}=p_{b_{i}}^{w}+q_{b_{i}}^{w}t_{u}^{b}+v_{b_{i}}^{w}dt-P_{m}^{w},$$

After solving for the distance ${\hat{d}}_{m,k}^{w}$ , according to Section 2.5.1, the distance and speed of the tag to each base station can be inversely calculated as follows:


(24) $$v_{m,k}=H_{m,k}X_{m,k-1}-{\hat{d}}_{m,k}^{w},\sigma^{2}=0.1^{2},$$

where, $H_{m,k}=\left[\begin{array}{cc} 1 & 0 \end{array}\right]$ , $X_{m,k-1}={\left[\begin{array}{cc} d & v \end{array}\right]}^{T}$ , $\sigma^{2}$ is the variance of ${\hat{d}}_{m,k}^{w}$ .

## 3 Research on robust factor graph based on sliding window real-time estimation of adaptive factor

The most significant deficiency in the factor graphs was the distribution of weights. Traditional factor graphs have serious problems with the distribution of observation information weights. This study introduces a robust estimation algorithm to solve this problem based on reliability, suppressing the influence of observation anomalies by changing the size of the observation noise covariance matrix.

### 3.1 Research of robust factor graph

In the VIU navigation system, the observations of other sensors produce abnormal observations owing to their own reasons or external influences, and these abnormal observations are gross errors. In actual system operation, if the gross error acts on the system, it will cause a deviation between the system measurement model and the actual measurement value, thus affecting the stability of optimization algorithm. Therefore, robust estimation is added to the algorithm, and its purpose is to improve the estimation accuracy by increasing the corresponding observation covariance matrix and reducing the reliability of the observation when the gross error of the observation is detected. To address these problems, this paper proposes a robust factor graph algorithm with dual functions of weight adjustment and gross error elimination. By changing the size of each sensor’s observation noise covariance matrix, the influence of observation anomalies was suppressed, and the positioning accuracy and robustness of the integrated navigation are improved.

**Figure 5:**
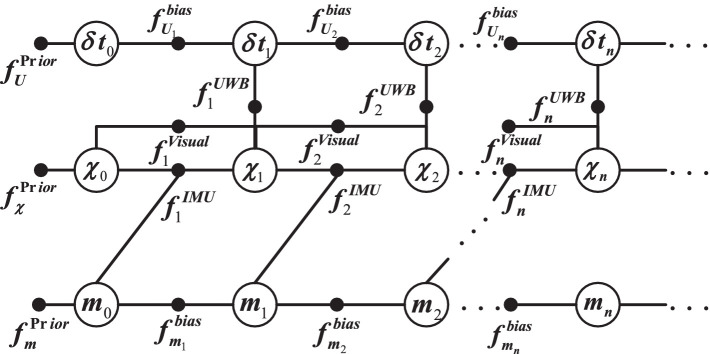
Global model factor for the VIU navigation system.

Figure 5 shows the global model factors for the VIU navigation system. Where $x_{n}$ represents the carrier navigation state variable at the nth time; $m_{n}$ represents the calibration parameters of the IMU (including constant drift and random walk terms, etc.) at the nth time, which are used to correct the data output by the IMU; the variable node set is denoted as χ ; the factor node set is denoted as F ; all edges connecting nodes form a set E , and the factor graph can be expressed as


(25) G=(F,χ,E),

According to factor graph theory, factor graph G describes the factorization of the function f(χ) , expressed as


(26) $$f\left(\chi \right)=\underset{i}{\prod }f\left(\chi_{i}\right),$$

In the multi-source information factor graph framework, a measurement model h(⋅) is defined, which can predict the observed information of the sensor based on the given state estimate. The factor node is defined as the difference between the predicted and actual measurements, and the corresponding indicator function is established to estimate the state variable. Based on the assumption of the Gaussian white noise model, a measurement factor node can be expressed as follows:


(27) $$f_{i}\left(\chi_{i}\right)=d\left[h_{i}\left(\chi_{i}\right)-z_{i}\right],$$

where $h_{i}\left(\chi_{i}\right)$ represents the measurement model; $z_{i}$ represents the actual observation information; d(⋅) represents the cost function.

According to the full probability Bayes formula, the state variable with the largest posterior probability density is considered the optimal estimate:


(28) $${\hat{\chi }}_{i}^{MAP}=\arg\max p\left(\chi_{i}|z_{i}\right),$$

For a complete factor graph system, the maximum *a posteriori* estimate of the joint probability density function is equivalent to minimizing the sum of the error equations for all nodes as follows:


(29) $${\hat{\chi }}_{i}^{MAP}=\arg\max p\left(\chi_{i}|z_{i}\right)=\arg\underset{V_{k}}{\min}\underset{i}{\sum }W\left(V_{k}\right)h\left(V_{k}^{i},z_{i}\right),$$

where, $W\left(V_{k}\right)$ is the weight function. The 3-stage method was used to construct the weight function as follows:


(30) $$W\left(V_{k}\right)=\{\begin{array}{c} \begin{array}{cc} 1, & \left|V_{k}\right|\leq k_{0} \end{array}\\ \begin{array}{cc} \frac{k_{0}}{\left|V_{k}\right|}\left(\frac{k_{1}-\left|V_{k}\right|}{k_{1}-k_{0}}\right), & k_{0}\leq \left|V_{k}\right|\leq k_{1} \end{array}\\ \begin{array}{cc} 10e-8, & \left|V_{k}\right|\geq k_{1} \end{array} \end{array},$$

$V_{k}$ is the prior residual at time k. Assume that the measurement covariance matrix before adjustment is $diag\left[\delta_{1},\delta_{2},\cdots ,\delta_{n}\right]$ and the covariance after adding the adaptive factor is $diag\left[\lambda_{1}\delta_{1},\lambda_{2}\delta_{2},\cdots ,\lambda_{n}\delta_{n}\right]$ . According to Eq. (30), the dynamic weight function $W\left(V_{k}\right)$ can be regarded as a generally decreasing function. The larger the measured and predicted values residuals, the smaller the assigned weights. The measurement was considered acceptable when the residual was smaller than a certain threshold. If the residual error between the sensor’s actual measurement value and the system state’s prediction is too large, it can be considered that the corresponding sensor is unreliable. Therefore, the trusted distance is so large that the fusion result does not depend on the measurement of the corresponding sensor, and the weight of the corresponding measurement is small and close to zero. When the trusted distance is greater than the threshold but not too large, the weight assigned to the corresponding sensor measurement information is dynamically adjusted according to the weight function. The specific operation is illustrated is shown in Appendix Figure A4.

The weight function is mainly set to the above form for the following considerations. First, the weight of each factor can be adjusted in real-time according to the residual. Second, in the case of sudden changes in vehicle motion, the measurement is prevented from being misjudged as an abnormal value owing to the significant deviation between the actual measurement and prediction of the system state. This not only provides the dual functions of weight adjustment and gross error removal but also enhances the robustness of the factor graph algorithm. This method differs from existing factor graph algorithms in that after all state variables are optimized, prediction residuals are computed before adding new metrics to the factor graph. The residual thresholds $k_{0}$ and $k_{1}$ define the credible range. We must set $V_{k}$ between the independent variable with the highest probability and the distribution mean to ensure that the probability of the credible probability is not too small and is statistically significant.

### 3.2 Real-time estimation of adaptive factor based on sliding window

Real-world navigation scenarios are complex and unpredictable. In other word, the real measurement noise is strongly dependent on the navigation scenarios. However, in many applications, it is difficult to predict the navigation environment. To solve this problem, the adaptive factor graph optimization is the most commonly-used method. However, this method is always an innovation sequence-based adaptive estimation approach and will involve the priori information χ during the calculation of the measurement noise covariance. Therefore, if the priori information is not well estimated, a negative effect properly occurs for the optimization performance. Hence, to avoid such risks, a novel adaptive model is proposed based on redundant measurement information. The specific derivation steps of the proposed model are as follows.

Assume that $z_{1}\left(k\right)$ and $z_{2}\left(k\right)$ are measurements of the value z from different systems at time k .


(31) $$\{\begin{array}{l} z_{P,1}\left(k\right)=z_{P}\left(k\right)-\left[f_{P,1}\left(k\right)+V_{P,1}\left(k\right)\right]\\ z_{P,2}\left(k\right)=z_{P}\left(k\right)-\left[f_{P,2}\left(k\right)+V_{P,2}\left(k\right)\right] \end{array}$$

where $V_{P,1}\left(k\right)$ and $V_{P,2}\left(k\right)$ are independent and zero mean white noises of UWB ranging; $f_{P,1}\left(k\right)$ and $f_{P,2}\left(k\right)$ are trend items of the measurement errors of UWB ranging.

First, calculate the difference sequence (i.e., the differences between every two adjacent measurements) of the two separate measurement systems:


(32) $$\left\{ \begin{array}{l} \Delta z_{P,1}\left(k\right)=z_{P,1}\left(k\right)-z_{P,1}\left(k-1\right)=z_{P}\left(k\right)-f_{P,1}\left(k\right)-V_{P,1}\left(k\right)-\\ \left(z_{P}\left(k-1\right)-f_{P,1}\left(k-1\right)-V_{P,1}\left(k-1\right)\right)\\ \Delta z_{P,2}\left(k\right)=z_{P,2}\left(k\right)-z_{P,2}\left(k-1\right)=z_{P}\left(k\right)-f_{P,2}\left(k\right)-V_{P,2}\left(k\right)-\\ \left(z_{P}\left(k-1\right)-f_{P,2}\left(k-1\right)-V_{P,2}\left(k-1\right)\right) \end{array}\right.$$

Then, subtract the two difference sequences and yield the second order difference sequences; the trend items $f_{P,1}$ and $f_{P,2}$ are extremely small values compared to the measurement noise, so they are neglected after subtraction:


(33) $$\begin{array}{l} \Delta z_{P,1}\left(k\right)-\Delta z_{P,2}\left(k\right)=\left[f_{P,1}\left(k-1\right)-f_{P,1}\left(k\right)\right]-\left[f_{P,2}\left(k-1\right)-f_{P,2}\left(k\right)\right]+\\ \left[V_{P,1}\left(k-1\right)-V_{P,1}\left(k\right)\right]-\left[V_{P,2}\left(k-1\right)-V_{P,2}\left(k\right)\right] \end{array}$$

(34) $$\begin{array}{l} \Delta z_{P,1}\left(k\right)-\Delta z_{P,2}\left(k\right)\approx \left[V_{P,1}\left(k-1\right)-V_{P,1}\left(k\right)\right]\\ \;\;\;\;\;\;\;\;\;\;\;\;\;\;\;\;\;\;\;\;\;\;\;-\left[V_{P,2}\left(k-1\right)-V_{P,2}\left(k\right)\right] \end{array}$$

Since $V_{P,1}\left(k\right)$ and $V_{P,2}\left(k\right)$ are uncorrelated, zero mean white noises, the auto-correlation of the second order difference sequences is:


(35) $$\begin{array}{l} \begin{array}{l} D\left(\left[\Delta z_{P,1}\left(k\right)-\Delta z_{P,2}\left(k\right)\right]\right)\\ =E\left\{\left[\Delta z_{P,1}\left(k\right)-\Delta z_{P,2}\left(k\right)\right]{\left[\Delta z_{P,1}\left(k\right)-\Delta z_{P,2}\left(k\right)\right]}^{T}\right\} \end{array}\\ \approx E\left\{V_{P,1}\left(k-1\right)V_{P,1}^{T}\left(k-1\right)\right\}+E\left\{V_{P,1}\left(k\right)V_{P,1}^{T}\left(k\right)\right\} \end{array}$$

When the prerequisite Eq. (5) is satisfied, the variance of measurement $z_{P,1}$ can be calculated as:


(36) $$R_{P,1}=D\left(V_{P,1}\left(k\right)\right)\approx D\left(\left[\Delta z_{P,1}\left(k\right)-\Delta z_{P,2}\left(k\right)\right]\right)/2$$

The precondition of the theorem is that two separate measurement systems are available for the same value z . This is suitable for the tightly-coupled system VIU, because UWB can provide the measurements of UWB ranging in a direct manner, and the Vision/IMU can provide them in an indirect approach. Hence, the UWB and Vision/IMU are treated as systems 1 and 2, respectively, in the proposed system.

On the other side, as the Vision/IMU owns the short-term accuracy characteristic, the errors that accumulated in several seconds are much smaller than the UWB ranging errors and, thus, can be neglected. Therefore, the tightly-coupled VIU also meets the prior condition in Eq. (33). Hence, the proposed method can be applied in the tightly-coupled VIU system to estimate the variances of the UWB ranging. Furthermore, a sliding window strategy is designed for noise estimation. The measurement noise is not always identically distributed and may change during the process; thus, using a sliding window can track the real-time noises accurately and mitigate the influence of historical information.


(37) $$R_{P,1}=D\left(V_{P,1}\left(k\right)\right)\approx D\left(\left[\Delta z_{P,1}\left(k-W:k\right)-\Delta z_{P,2}\left(k-W:k\right)\right]\right)/2$$

where k denotes the current time epoch and W denotes the size of the sliding window, which is usually set as 10–20. To improve the efficiency of using current observation information and reduce the contribution of historical parameters to current state parameters, the exponential expansion method is used as follows:


(38) $$\begin{array}{l} R_{P,1}=D\left(V_{P,1}\left(k\right)\right)\\ \approx D\left(e^{\left(t_{k}-t_{k-W}\right)/\tau }\left[\Delta z_{P,1}\left(k-W:W\right)-\Delta z_{P,2}\left(k-W:W\right)\right]\right)/2 \end{array}$$

where, τ is the UWB sampling interval. The standard deviation $V_{k}$ can be written as:


(39) $$V_{k}=\sqrt{R_{P,1}}$$

## 4 Multi-scenes experimental verification

In this section, we describe an experiment conducted to test the performance of the proposed method in a vehicle experiment using a tightly coupled VIU navigation system. The UWB adopts products released by Noop-loop manufacturers, and the ranging accuracy is approximately 5 cm under LOS conditions. The speed is relatively low; therefore, the influence of asynchrony among the base stations can be ignored.

The camera adopts the standard version of Mynteye’s mono-inertial camera, and the IMU adopts a 6-axis system (3-axis accelerometer +3-axis gyroscope) that comes with Mynteye’s mono-inertial camera. A specific experimental setup is shown in Appendix Figure A5A. The data collected by the infrared motion capture camera are used as the true value reference, and the accuracy can generally reach the millimeter level. The IMU parameters are listed in Appendix Table B1. The collected accelerometer and gyroscope data are connected to TX2 through the UWB 3.0 interface and published to the ROS platform so that each program can access the measurement data.

We used three different datasets to compare RFG-TVIU with three other models, including FG-VIO (VINS-Mono without loop), IMU/UWB (UWB hardware’s own IMU/UWB fusion algorithm, a relatively stable UWB localization algorithm) and FG-TVIU (VIU with tightly coupled based on factor graph) through several different scenes and present the comparative analysis results, and provided comparative analysis results. In this study, the RMSE can be defined as


(40) $$RMSE=\sqrt{\frac{1}{N}\overset{N}{\underset{k=1}{\sum }}{\left(V_{k}\right)}^{2}},$$

where N denotes the total sample number, $V_{k}$ represents the difference between the reference value and the sampled value at time k. RMSE is used to reflect the deviation between the estimated value and the reference value, and it is very sensitive to large errors in a set of data. Therefore, we used the RMSE to evaluate the influence of outliers on the localisation results.

(1) Scenes 1-1, 1-2, and 3 were performed in an open environment with UWB under LOS conditions, and the experiment took approximately 150 s.
(2) Scenes 2-1, 2-2, and 2-3 were performed in an indoor environment where the UWB signal was weak, and NLOS errors were present. The NLOS scene was mainly imitated by the occlusion of three large plastic wooden boards. A specific scenario is shown in Appendix Figure A5B. The experiment for each scene lasted for approximately 120 s.
(3) Scenes 3-1 and 3-2 were performed in an outdoor environment on the playground of Southeast University, which is used for conducting ablation experiments.

Scene 1 and Scene 2 belong to indoor scenes, and their true values are collected by infrared motion capture cameras; Scene 3 belongs to an outdoor scene, and the true values are collected from the GNSS antenna.

### 4.1 Comparison of performance among different schemes under the condition of UWB LOS in scenes 1-1 and 1-2

The raw data analysis of the IMU and UWB in Scene 1-1 is shown in Appendix Figure A6. The IMU includes 3-axis accelerometer and a gyroscope angular velocity. The top two in Appendix Figure A6 are the acceleration and angular velocity, and the bottom two are the UWB original data and adjacent time differential data. Due to the similarity between Scenes 1-1 and 1-2, we will only analyze Scene 1-1 below.

Given that the IMU is in the horizontal direction, the acceleration value in the X (vertical direction) direction fluctuates around 10 m/s^2^, and the angular velocity values in the Y and Z directions fluctuate around 0. Nguyen et al. (2021) shows that when the original data of UWB fluctuates significantly, for example, when NLOS occurs, it can be detected by the difference in adjacent time data. It can be seen from Appendix Figure A6 that, except for a few moments, the UWB data are stable. Table 1 lists the root mean square errors (RMSE) of the position errors obtained using different methods.

**Table 1:** The RMSE results on Scenes 1-1 and 1-2.

Algorithm type	Scene 1-1 (cm)	Increase (%)	Scene 1-2 (cm)	Increase (%)
FG-VIO	22.6	-	24.9	-
IMU/UWB	12.7	43.36	13.5	45.78
FG-TVIU	10.1	55.75	10.4	58.23
RFG-TVIU	**8.4**	**62.83**	**8.9**	**64.26**

The translation (m) errors are listed as follows. The numbers in bold represent the estimated trajectory that is more close to the benchmark trajectory. Given that the reference of the rotation of Scene 1-1 and Scene 1-2 cannot be collected, we only give the comparison of translation.

#### 4.1.1 Trajectory and RMSE comparison of Scenes 1-1

**Figure 6:**
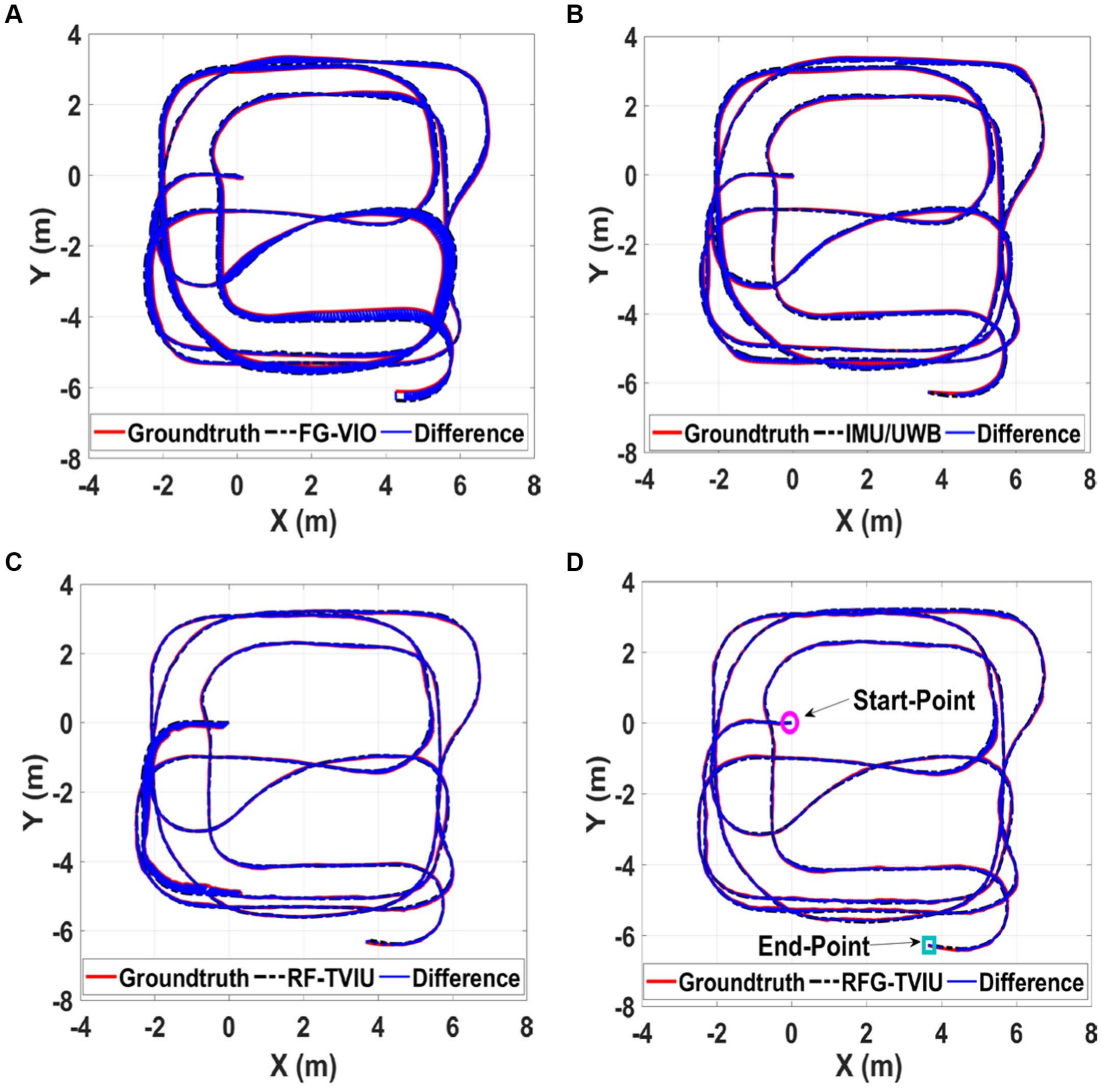
Comparison of the proposed method RFG-TVIU **(D)** versus FG-VIO **(A)**, IMU/UWB **(B)** and FG-TVIU **(C)** on the Scene 1-1 sequence. The blue line denotes the localization error. Quantitative results can be found in Table 1. It can be seen that our method produces better localization accuracy.

**Figure 7:**
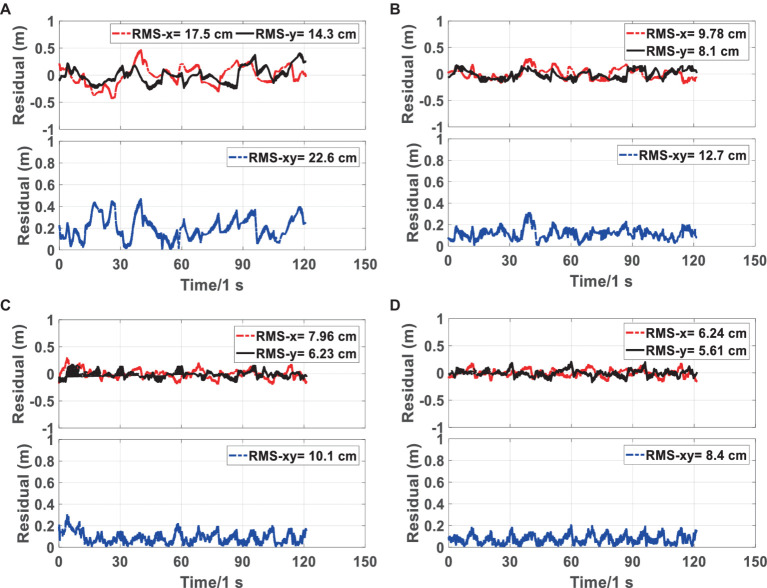
RMSE of the proposed method RFG-TVIU **(D)** versus FG-VIO **(A)**, IMU/UWB **(B)** and FG-TVIU **(C)** on the Scene 1-1 sequence. The blue line denotes the localization error. The red and black lines represent the residuals of the X and Y axes, respectively. Quantitative results can be found in Table 1. We can see that our method produces better localization accuracy.

Figures 6, 7 show the trajectory and RMSE comparison of the four schemes in Scene 1-1, respectively. The percent increase in RMSE was calculated as the percent increase in RMSE = (a-b)/a.

First, we compare the advantages and disadvantages of scheme 1 with other schemes. Since scheme 1 is based on VIO positioning, the error accumulates over time. As shown in Figures 6, 7, schemes 2, 3 and 4 are far better than scheme 1, compared with scheme 1, which is improved by 43.36, 55.75, and 62.83%, respectively.

We then compare the advantages and disadvantages of the other three schemes. Figure 7 and Table 1 show that the RMSE of scheme 4 is the smallest, and the error is maintained within 0.2 m. The scheme 3 is the second best, and scheme 2 is the worst. Given that schemes 3 and 4 are optimized based on the factor graphs, it can be seen from Table 1 that they are better than scheme 2. The scheme 4 has added a robust equivalent weight algorithm based on scheme 3, which can allocate the weight of the original UWB ranging more reasonably; therefore, it is more stable than scheme 3.

#### 4.1.2 Analysis of UWB pre-test and post-test residuals of Scene 1-1

The size of the residuals reflected the quality of the observed data. Generally, we can analyze the quality of the observed data by outputting the residual. The residuals include pre- and post-optimization residuals. The pre-optimization residual reflects the degree of consistency between the prior prediction of the system and the current measurement, and the degree of consistency between the current optimization model and the current observation of the post-test residual. The difference between the pre-optimization and post-optimization residuals reflects the accuracy of the system algorithm.

The pre- and post-optimization residuals of each UWB base station in Scene 1-1 is shown in Appendix Figure A7. It can be seen from the Appendix Figure A7 that after the system converges, the pre-optimization and post-optimization residuals fluctuate between −0.1 m and 0.1 m, which is basically the same as the variation of the ranging noise of UWB. Moreover, the changes in the pre-and post-optimization residuals are basically the same, which indicates that the algorithm model and observation quality in this study are relatively good.

### 4.2 Comparison of performance among different schemes under the condition of UWB NLOS in Scenes 2-1, 2-2, and 2-3

The schematics of Scenarios 2-1, 2-2, and 2-3 are shown in Appendix Figure A8. Three baffles were placed in the scenarios to simulate a complex environment. One baffle (see Appendix Figure A5B) was placed in Scene 2-1, and two and three baffles were placed in Scenarios 2-2 and 2-3, respectively, as shown in Appendix Figure A8. The combined system moved back and forth between the three baffles. We can use infrared motion capture cameras to obtain the true values of trajectories.

**Figure 8:**
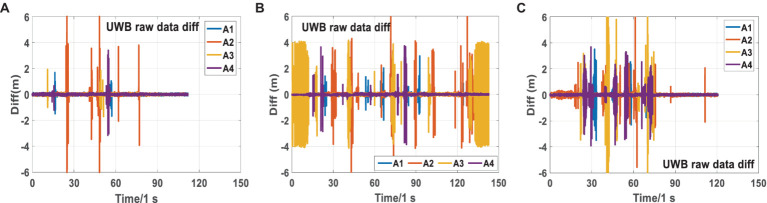
Analysis of UWB raw differential ranging NLOS errors in Scenarios 2-1 **(A)**, 2-2 **(B)**, and 2-3 **(C)**.

Figure 8 shows the original UWB differential ranging analysis of the three scenarios in scene 2. It can be seen from Figure 8 that there are relatively serious NLOS phenomena in the three scenarios, especially in Scenarios 2-2 and 2-3. NLOS lasted for a long time in the three scenarios, almost throughout the observation stage. During many periods, four base stations had serious NLOS errors at the same time. Compared to Scenarios 2-2 and 2-3, the NLOS phenomenon of Scene 2-1 is relatively slight.

#### 4.2.1 Trajectory and RMSE comparison of Scenarios 2-1, 2-2, and 2-3

The trajectories and RMSE comparison of different schemes for the three scenarios in Scene 2 are shown in Appendix Figures A9–A16. In the RMSE comparison, the blue line represents the positioning error, and the red and black lines represent the errors on the X- and Y-axes, respectively. The quantitative results are presented in Table 2. The red numbers indicate that the percentage of the algorithm decreases relative to that of FG-VIO. To compare the advantages of this study more vividly, we join the tight combination IMU/UWB algorithm that comes with NOOP-LOOP.

**Table 2:** Comparison of RMSE of Scenarios 2-1, 2-2 and 2-3.

Type	Scene 2-1 (cm)	Improve (%)	Scene 2-2 (cm)	Improve (%)	Scene 2-3 (cm)	Improve (%)
FG-VIO	62.2	-	56.6	-	42.5	-
IMU/UWB	28.3	54.50	67.8	−19.79	89.4	−110.35
FG-TVIU	26.5	57.40	57.6	−1.77	76.9	−80.94
RFG-TVIU	**11.1**	**82.15**	**16.8**	**70.32**	**10.5**	**75.29**

From the RMSE and trajectory comparison in the three scenarios, it can be observed that RFG-TVIU produces better localization accuracy. Given that the positioning error of IMU/UWB is generally larger than that of FG-TVIU in NLOS scenarios, a comparative analysis of FG-VIO, FG-TVIU and RFG-TVIU is performed below.

First, we analyzed FG-VIO and FG-TVIU. The Appendix Figures A9–A12 show that in Scenarios 2-2 and 2-3, due to serious NLOS errors, the positioning accuracy of the combined system is seriously affected. Owing to the influence of NLOS errors, the accuracy worsens, and the RMSE of FG-TVIU is greater than that of FG-VIO. In particular, for Scene 2-3, the maximum error can reach 6 m. In Scene 2-1, the NLOS error of the UWB ranging was relatively small, and the positioning accuracy of FG-TVIU was better than that of FG-VIO.

Then we analyzed FG-VIO and RFG-TVIU. The conclusion can be drawn from Appendix Figures A9–A12 that RMSE of RFG-TVIU in the three scenes is much smaller than that of FG-VIO and FG-TVIU. Compared with FG-VIO, the RMSE of RFG-TVIU in the three scenes is improved by more than 70%, the plane error is within 0.2 m after convergence, and both Scenes 2-1 and 2-3 are within 0.1 m. Table 2 shows that the NLOS error impacts the filter-based combination system more than the factor graph-based optimization. After adding the robust model, the NLOS error has little effect on the positioning accuracy of the combined system. In Scene 2-3, although there is a severe NLOS phenomenon in this scene, the added robust model has characteristics of weight adjustment and gross error elimination simultaneously, which can make better use of the ranging information of the UWB.

It can be observed from the above analysis that when one baffle is placed in the scene, although the positioning performance of FG-TVIU is also affected, the impact is relatively small, and the positioning accuracy of FG-TVIU is still better than that of FG-VIO. When two or three baffles are placed, the positioning performance of the FG-TVIU is significantly affected by the NLOS error, and the positioning accuracy decreases rapidly. However, the positioning performance of the RFG-TVIU is not affected by the NLOS error, and the accuracy can still reach approximately 10 cm. The UWB NLOS error for each moment is further analyzed below.

#### 4.2.2 Analysis of UWB pre-optimization and post-optimization residuals of Scenes 2-1, 2-2, and 2-3

**Figure 9:**
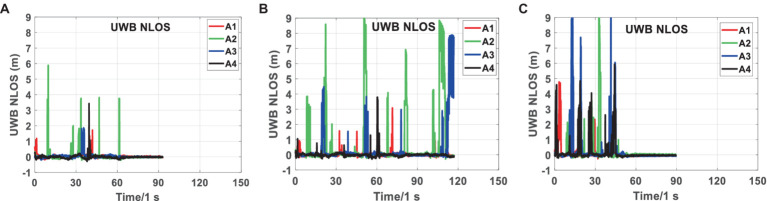
NLOS errors of UWB ranging on Scenarios 2-1 **(A)**, 2-2 **(B)**, and 2-3 **(C)** Sequences.

Figure 9 shows the real-time NLOS errors of tags to UWB base stations in Scenarios 2-1, 2-2 and 2-3. By comparing Figure 9 and Appendix Figure A7, we can see that owing to the three NLOS scenarios included in Scene 2, UWB ranging has a more severe NLOS phenomenon, which is more complicated than in Scene 1.

Given that the estimation of UWB base station parameters is already relatively stable, we can consider it as a known value when analyzing NLOS errors. When the posterior coordinate information of the UWB tag is relatively accurate, the NLOS error in the range of each base station can be calculated using Eq. (23).

According to Appendix Figure A7, when the NLOS error is not included in the ranging, the ranging residual fluctuates around the positive and negative 0 values after optimization, and the maximum fluctuation range is 0.2 m. Given that the NLOS error is semi-random, when the NLOS error is included in the range if the robust model is accurate enough, the main part of the NLOS error is included in the post-optimization residual. As shown in Figure 9, the NLOS errors in the three scenarios reached a maximum of 10 m. From Figure 9, we can see that the NLOS error from the tag to the same UWB base station can last for 5–10s. In the short term, the NLOS error of UWB base stations is relatively stable, which also verifies the effectiveness of the algorithm model in this paper. In addition, through comparison, we can also draw the following conclusion: when a robust model is added and the robust model is accurate enough, the UWB ranging error is mainly reflected in the posterior residual. When robust models are not added to tightly combined systems, the non-line of sight error of UWB is generally allocated to various parameters and posterior residuals.

### 4.3 Comparison of different UWB initial calibration schemes in Scene 3

When conducting VIU tight coupling, there are two schemes based on the initial calibration mode of UWB base stations: pre-calibration of UWB base stations and real-time estimation of UWB base stations as parameters. Scheme 1 requires us to use other equipment (such as a total station) to measure the coordinates of the UWB base station in advance, and to perform trajectory calibration in advance during fusion to solve for the rotation and translation matrices of the UWB base station coordinate system and the VIO initial world coordinate system. This mode not only limits the usage scenarios (such as when UWB needs to be moved in real-time), but also requires additional devices to assist in completing. Scheme 2 is more flexible compared to Option 1. By using UWB base stations as parameters for real-time estimation, it is possible to directly use UWB base station coordinates for global constraints without prior calibration and conversion during tight coupling. This study adopts Scheme 2 for tightly coupled research. The trajectory comparison and error comparison of the two schemes are shown in Appendix Figures A17, A18, respectively.

Comparing Appendix Figures A17A,B, it can be seen that at the initial time of 0–13 s, the accuracy of Scheme 2 is better than that of Scheme 1. After analysis, it can be concluded that due to the lack of pre calibration of the UWB base station in Scheme 1, real-time parameter estimation is required. Scheme 1 has more parameters to be estimated than Scheme 2, and the model structure is not as stable as Scheme 2. Therefore, Scheme 1 converges more slowly than Scheme 2 at the initial time. But after 13 s, the convergence of Schemes 1 and 2 tends to stabilize, and the results of Scheme 1 are better than those of System 2. The UWB base station in Scheme 1 is estimated in real-time as a parameter during initialization, so the coordinates of the base station are consistent with the initialized world coordinate system and there is no conversion problem. Therefore, after the UWB base station coordinate parameters converge in Scheme 1, the accuracy of system pose estimation is better than that of Scheme 2 estimation.

### 4.4 Ablation experiment

The effectiveness of the VIU tightly coupled model and the robust adaptive model were validated through ablation experiments, and the results are shown in Table 2. “Node1” in Table 3 refers to using only the original distance to participate in constraints in factor graph based VIU compact combinations; “Node2” refers to the distance difference participation constraint based on adjacent moments; “KF” refers to the addition of UWB raw ranging Kalman filtering module; “Robust” refers to the addition of an adaptive robust model in tight combinations.

**Table 3:** Results of ablation experiment (cm).

Scheme	Node1	Node2	KF	ROBUST	Dataset 3-1		Dataset 3-2	
					RMSE	Percentage	RMSE	Percentage
Model 1	√				9.9	0	14.0	0
Model 2	√	√			9.4	+0.5	12.6	+1.4
Model 3	√	√	√		9.1	+0.8	12.1	+1.9
Model 4	√	√	√	√	8.2	+1.7	10.8	+3.2

**Figure 10:**
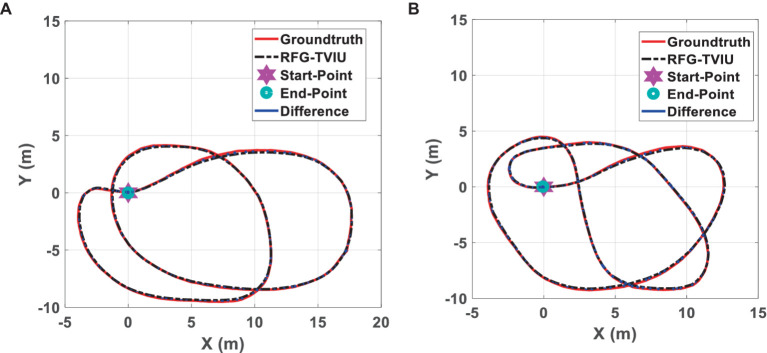
Trajectory of RFG-TVIU for Scenes 3-1 **(A)** and 3-2 **(B)**.

A real testing scene for dataset 3 is shown in Appendix Figure A19, located on the Sipailou playground of Southeast University. Figure 10 shows the test trajectory calculated by this scheme for scenes 3-1 and 3-2, and the mean error calculated by this scheme for scenarios 3-1 and 3-2 is shown in Appendix Figure A20. From the error chart, we can see that the mean error of both scenes is around 10 cm, and the error is relatively uniform with little significant fluctuation.

**Figure 11:**
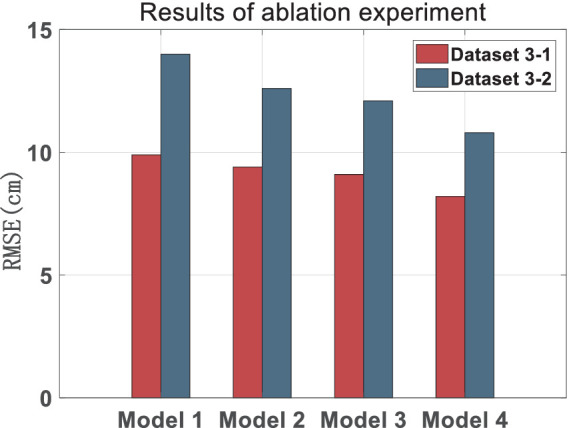
Results of ablation experiment.

Scenes 3-1 and 3-2 both show some improvement in accuracy compared to Model 1, with accuracy of 0.5 cm and 1.4 cm, respectively. In contrast, Scene 3-2 shows a greater improvement in accuracy. Similarly, in Scenes 3-1 and 3-2, Model 3 has improved by 0.8 mm and 1.9 mm compared to Model 1, respectively. The KF module only performs smoothing filtering on the raw UWB ranging, so the improvement is relatively small. Model 4 has improved by 1.7 mm and 3.2 mm compared to Model 1, respectively. We can see that the addition of Node 2, KF and ROBUST models have significantly improved its accuracy, but overall, the accuracy of scene 3-1 is higher than that of scene 3-2. Through analysis, it can be concluded that due to the presence of dynamic scenes and complex trajectories in scene 3-2, the overall accuracy is not as good as in scene 3-2. Meanwhile, due to the fact that Node 2 and ROBUST models are specifically designed to resist gross errors, the accuracy improvement in scene 3-1 is smaller than that in Scene 3-2. The specific comparison of the ablation experiment can be seen in Figure 11.

## 5 Conclusion

This paper proposes a “plug and play” VIU multi-sensor tightly-coupled system based on robust factor graph. The difference from traditional UWB-based tightly-coupled models is that the VIU tightly-coupled model in this study uses UWB base station coordinates as parameters for real-time estimation without pre-calibration. This study also proposes a novel adaptive robust factor graph model to solve the serious problem of traditional factor graph in the weight distribution of observation information.

Through a comparative analysis of Scenes 1 and 2, we can see that RFG-TVIU has a great improvement compared with other VIU methods that are tightly coupled. When the NLOS error of the UWB ranging is large, the positioning accuracy of the FG-TVIU decreases rapidly. RFG-TVIU is hardly affected by the NLOS error, and the RMSE can still reach approximately 10 cm. Even if four base stations have significant NLOS errors simultaneously, RFG-TVIU can ensure the output of high-precision positioning results. Comparing the localization RMSE in several scenarios, we can draw a conclusion that UWB NLOS has a more significant impact on the filter than the factor graphs. Compared with the algorithm based on the standard factor graph, the larger the NLOS error of UWB, the more pronounced the improvement in RFG-TVIU. It can be seen from the three scenarios in Scene 2 that the NLOS error is transient and systematic, and the rational use of this characteristic in back-end optimization can improve the accuracy and robustness of the fusion system. The effectiveness of each module proposed in this study has been demonstrated through ablation experiments.

Although increasing the UWB base station parameters for estimation reduces accuracy in the initial stage, as the UWB base station coordinate system is consistent with the world coordinate system and does not require coordinate conversion, the accuracy after system convergence is better than a tightly combined system with UWB base station coordinate pre-calibrated. Next, we will study tight combination localization under dynamic UWB base stations.

## Data availability statement

The original contributions presented in the study are included in the article/Supplementary material, further inquiries can be directed to the corresponding author.

## Author contributions

GF: Data curation, Formal analysis, Project administration, Visualization, Writing – original draft. QW: Funding acquisition, Methodology, Visualization, Writing – review & editing. GY: Data curation, Methodology, Visualization, Writing – review & editing. PL: Data curation, Methodology, Visualization, Writing – review & editing.

## References

[ref1] Bresson G. Rahal M. Gruyer D. Revilloud M. Alsayed Z. (2016). A cooperative fusion architecture for robust localization: application to autonomous driving. IEEE 19th international conference on intelligent transportation systems.

[ref2] Chang L. Niu X. Liu T. (2020). GNSS/IMU/ODO/LiDAR-SLAM integrated navigation system using IMU/ODO pre-integration. Sensors 20:4702. doi: 10.3390/s20174702 32825329 PMC7506683

[ref3] Chen C. Zhu H. Li M. You S. (2018). A review of visual-inertial simultaneous localization and mapping from filtering-based and optimization-based perspectives. Robotics 7:45. doi: 10.3390/robotics7030045

[ref4] Cheng J. Kim J. Jiang Z. Zhang W. (2014). Tightly coupled SLAM/GNSS for land vehicle navigation. Lect. Notes Electr. Eng. 305, 721–733. doi: 10.1007/978-3-642-54740-9_64

[ref5] Dong X. Gao Y. Guo J. Zuo S. Xiang J. Li D.. (2022). Approaching and landing of UAVs. Aerospace 9:797. doi: 10.3390/aerospace9120797

[ref6] Du S. Q. (2012). A non-smooth Levenberg-Marquardt method for generalized complementarity problem. J. Inform. Comput. Sci. 7, 267–271.

[ref7] Gao W. Meng X. Gao C. Pan S. Wang D. (2017). Combined GPS and BDS for single-frequency continuous RTK positioning through real-time estimation of differential inter-system biases. GPS Solutions 22:20.

[ref8] Guowei W. Xiaolong Y. Cai R. Li H. Wang H. Song S.. (2018). Robust and precise vehicle localization based on multi-sensor fusion in Diverse City scenarios. IEEE international conference on robotics and automation (ICRA), Brisbane, Australia.

[ref9] Hu C. Huang P. Wang W. (2023). Tightly coupled visual-inertial-UWB indoor localization system with multiple position-unknown anchors. IEEE Robot. Automat. Lett.

[ref10] Indelman V. Williams S. Kaess M. Dellaert F. (2013). Information fusion in navigation systems via factor graph based incremental smoothing. Robotics Auton. Syst. 61, 721–738. doi: 10.1016/j.robot.2013.05.001

[ref11] Kao P. Y. Chang H. J. Tseng K. W. Chen T. Luo H. L. Hung Y. P. (2023). VIUNet: deep visual–inertial–UWB fusion for indoor UAV localization. IEEE. Access 11, 61525–61534. doi: 10.1109/ACCESS.2023.3279292

[ref12] Li X. Wang Y. (2021). Research on a factor graph-based robust UWB positioning algorithm in NLOS environments. Telecommun. Syst. 76, 207–217. doi: 10.1007/s11235-020-00709-2

[ref13] Li T. Zhang H. Gao Z. Niu X. El-sheimy N. (2019). Tight fusion of a monocular camera, MEMS-IMU, and single-frequency multi-GNSS RTK for precise navigation in GNSS-challenged environments. Remote Sens. 11. doi: 10.3390/rs11060610

[ref14] Liu T. Li B. Chen G. Yang L. Qiao J. Chen W. Tightly coupled integration of GNSS/UWB/VIO for reliable and seamless positioning. IEEE Trans. Intell. Transport. Syst 25, 2116–2128. doi: 10.1109/TITS.2023.3314836

[ref15] Mascaro R. Teixeira L. Hinzmann T. Siegwart R. Chli M. (2018). GOMSF: graph-optimization based multi-sensor fusion for robust UAV pose estimation. 2018 IEEE international conference on robotics and automation (ICRA): 1421–1428.

[ref16] Mikhail S. Abhinav R. Han-Pang C. Kevin K. Supun S. David P. S. (2019). Multi-sensor fusion for motion estimation in visually-degraded environments. IEEE international symposium on safety, security, and rescue Robotics, pp: 7–14.

[ref17] Mourikis A. I. (2007). A multi-state constraint Kalman filter for vision-aided inertial navigation. IEEE International Conference on Robotics and Automation (ICRA). Rome, Italy. 3565–3572.

[ref18] Nguyen T. H. Nguyen T. -M. Xie L. (2021). Range-focused fusion of camera-IMU-UWB for accurate and drift-reduced localization. IEEE Robot. Automat. Lett. 6, 1678–1685. doi: 10.1109/LRA.2021.3057838

[ref19] Ochoa-de-Eribe-Landaberea A. Zamora-Cadenas L. Peñagaricano-Muñoa O. Velez I. (2022). UWB and IMU-based UAV’s assistance system for autonomous landing on a platform. Sensors 22:2347. doi: 10.3390/s22062347 35336532 PMC8948988

[ref20] Patrick G. Kevin E. Guoquan H. (2018). Asynchronous multi-sensor fusion for 3DMapping and localization. IEEE International Conference on Robotics and Automation.

[ref21] Paul G. Kyle O. (2018). Tightly-coupled GNSS/vision using a sky-pointing camera for vehicle navigation in urban areas. Sensors 18:1244. 29673230 10.3390/s18041244 PMC5948580

[ref22] Qin Tong L. I. Peiliang L. Shaojie S. H. E. N. (2017). VINS-Mono: A Robust and Versatile Monocular Visual-Inertial State Estimator. IEEE Trans. Robot. 99:1.

[ref23] Schreiber M. Königshof H. Hellmund A.-M. Stiller C. (2016). Vehicle localization with tightly coupled GNSS and visual Odometry. IEEE Intel. Vehicles Symp.

[ref24] Shao W. Z. Srinivasan V. Cong L. George K. (2019). Stereo visual inertial LiDAR simultaneous localization and mapping. IEEE International Conference on Intelligent Robots and Systems. pp: 370–377.

[ref25] Song Y. Guan M. Tay W. P. Law C. L. Wen C. (2019). UWB/LiDAR fusion for cooperative range-only SLAM. IEEE International Conference on Robotics and Automation (ICRA), pp. 6568–6574.

[ref26] Suhr J. Jang J. Min D. Jung H. (2017). Sensor fusion-based low-cost vehicle localization system for complex urban environments. IEEE Trans. Intell. Transp. Syst. 18, 1078–1086. doi: 10.1109/TITS.2016.2595618

[ref27] Usenko V. Engel J. Stuckler J. Cremers D. (2016). Direct visual-inertial odometry with stereo cameras. 2016 IEEE international conference on robotics and automation (ICRA): 1885–1892.

[ref28] Wei X. Li J. Zhang D. Feng K. (2021). An improved integrated navigation method with enhanced robustness based on factor graph. Mech. Syst. Signal Process. 155:107565. doi: 10.1016/j.ymssp.2020.107565

[ref29] Xie J. He X. Mao J. Zhang L. Hu X. (2022). C2VIR-SLAM: centralized collaborative visual-inertial-range simultaneous localization and mapping. Drones 6:312. doi: 10.3390/drones6110312

[ref30] Xu C. Liu Z. Li Z. (2021). Robust visual-inertial navigation system for low precision sensors under indoor and outdoor environments. Remote Sens. 13:772.

[ref31] Yang Z. Shen S. (2017). Monocular visual–inertial state estimation with online initialization and camera–IMU extrinsic calibration. IEEE Trans. Automat. Sci. Eng. 14, 39–51.

[ref32] Yang G. Wang Q. Liu P. Yan C. (2021). PLS-VINS: visual inertial state estimator with point-line features fusion and structural constraints. IEEE Sensors J. 21, 27967–27981. doi: 10.1109/JSEN.2021.3123973

[ref33] Zhang Y. L. Tan J. D. Zeng Z. M. Liang W. Xia Y. (2014). Monocular camera and IMU integration for indoor position estimation. Conference proceedings: Annual International Conference of the IEEE Engineering in Medicine and Biology Society. IEEE Engineering in Medicine and Biology Society. Annual Conference. 10.1109/EMBC.2014.6943811 25570179

[ref34] Zheng G. Ling P. Zou D. Miao R. Liu P. Yu W. (2016). Graphical approach for MAV sensors fusion. The 29th international technical meeting of the satellite division of the Institute of Navigation.

