# Supplementary material for: RFG-TVIU: robust factor graph for tightly coupled vision/IMU/UWB integration
Source: Front Neurorobot. 2024 Apr 29;18:1343644. doi: 10.3389/fnbot.2024.1343644 (PMC11089196; doi:10.3389/fnbot.2024.1343644)
Supplement: Supplementary file 1 [file Data_Sheet_1.docx]

Figure A1 The diagram of VIU with tightly coupled

Figure A2 Vision constraint factor-graph construction

Figure A3 UWB differential ranging constraints

Figure A4 Weight adjustment graph


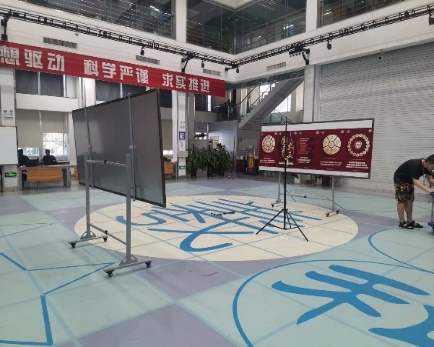


(a) (b)

Figure A5 VIU tight combination experimental scene diagram (a) and Deployment of UWB NLOS in Scenes 2-1, 2-2 and 2-3 (b)

Figure A6 IMU and UWB raw data analysis in Scene 1-1

Figure A7 UWB pre-test and post-test residual in Scene 1-1

|  | | |
| --- | --- | --- |
| (a) | (b) | (c) |

Figure A8 Layout of complex Scenarios in Scenarios 2-1(a), 2-2(b) and 2-3(b)

|   (a) |   (b) |   (c) |
| --- | --- | --- |

Figure A9 Trajectory comparison of FG-VIO for Scene 2-1(a), Scene 2-2(b) and Scene 2-3(b)

|   (a) |   (b) |   (c) |
| --- | --- | --- |

Figure A10 Trajectory comparison of IMU/UWB for Scene 2-1(a), Scene 2-2(b), and Scene 2-3(b)

|   (a) |   (b) |   (c) |
| --- | --- | --- |

Figure A11 Trajectory comparison of FG-TVIU for Scene 2-1(a), Scene 2-2(b) and Scene 2-3(b)

|   (a) |   (b) |   (c) |
| --- | --- | --- |

Figure A12 Trajectory comparison of RFG-TVIU for Scene 2-1(a), Scene 2-2(b) and Scene 2-3(b)

|   (a) |   (b) |   (c) |
| --- | --- | --- |

Figure A13 RMSE comparison of FG-VIO for Scene 2-1(a), Scene 2-2(b) and Scene 2-3(b)

|   (a) |   (b) |   (c) |
| --- | --- | --- |

Figure A14 RMSE comparison of FG-VIO for Scene 2-1(a), Scene 2-2(b) and Scene 2-3(b)

|   (a) |   (b) |   (c) |
| --- | --- | --- |

Figure A15 RMSE comparison of FG-TVIU for Scene 2-1(a), Scene 2-2(b) and Scene 2-3(b)

|   (a) |   (b) |   (c) |
| --- | --- | --- |

Figure A16 RMSE comparison of RFG-TVIU for Scenarios 2-1(a), 2-2(b) and 2-3(b)

| 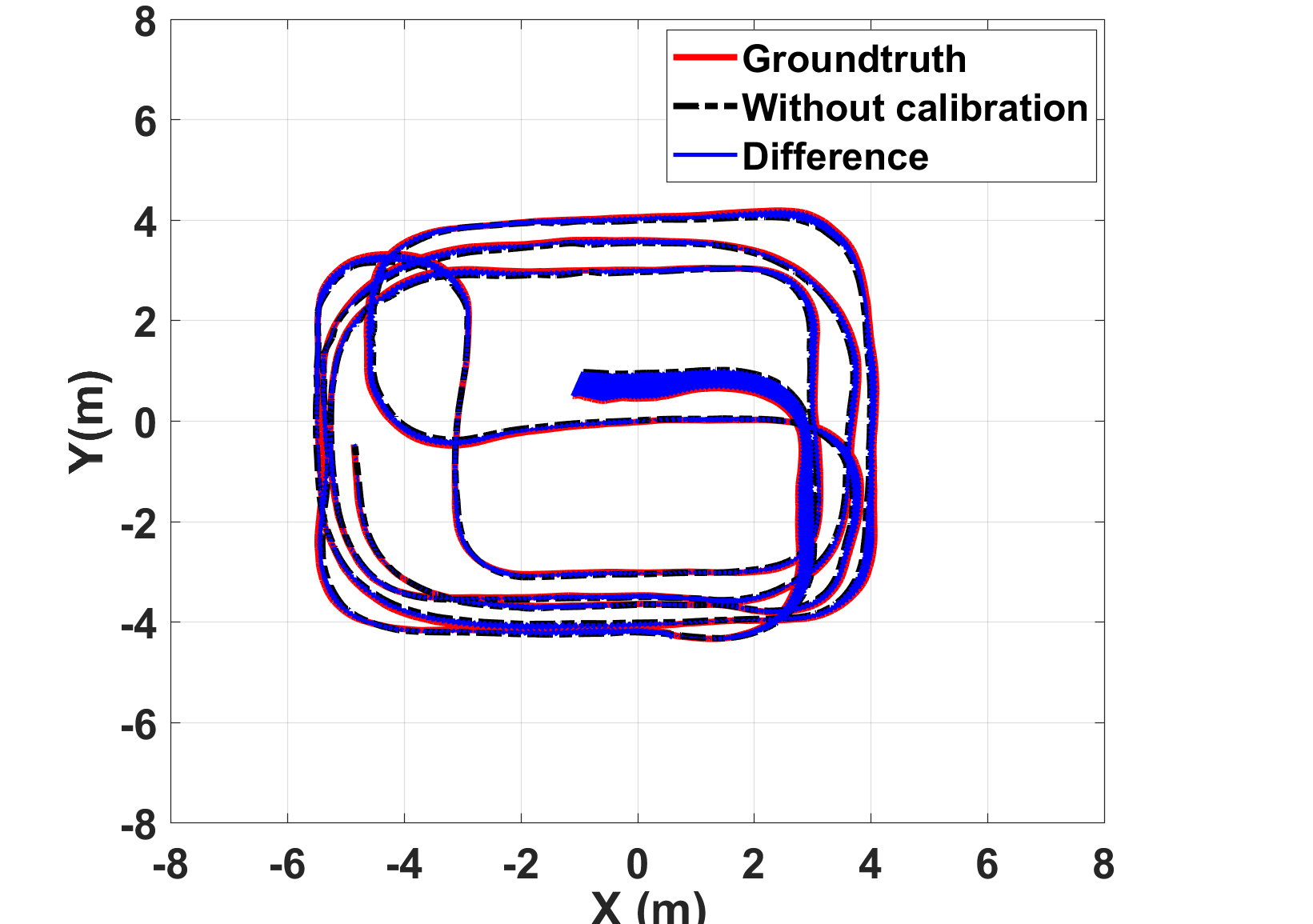  (a) | 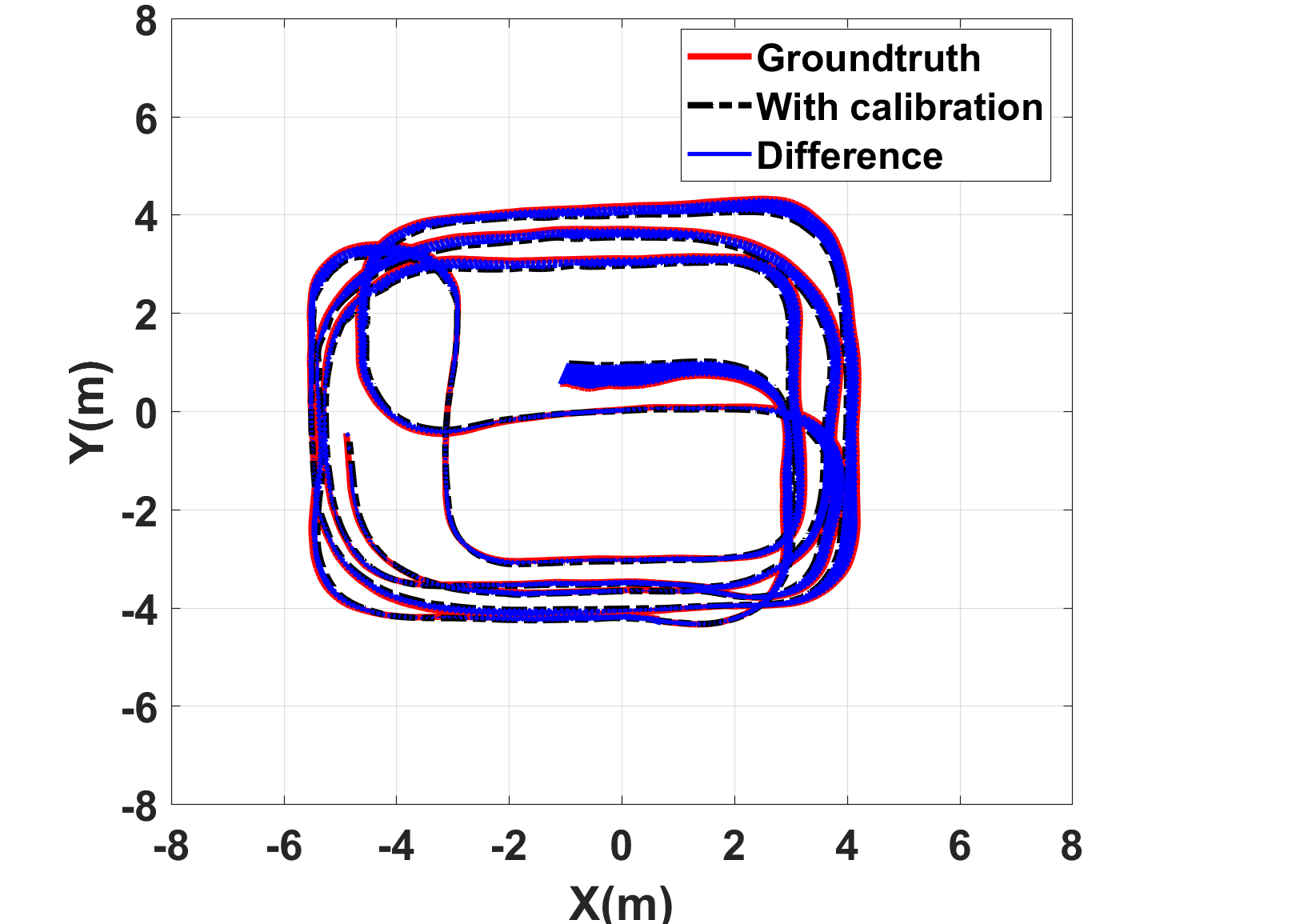  (b) |
| --- | --- |

Figure A17 Trajectory comparison of FG-VIO for Scene 2-1(a), Scene 2-2(b) and Scene 2-3(b)

|   (a) |   (b) |
| --- | --- |

FigureA18 RMSE comparison of RFG-TVIU for Scenarios 2-1(a), 2-2(b) and 2-3(b)

| 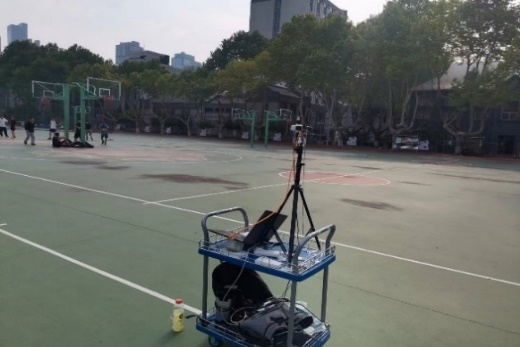  (a) | 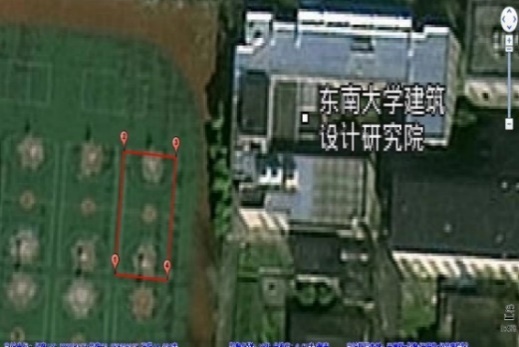  (b) |
| --- | --- |

Figure A19 Scene 3 actual scene map (a) and scene grid map (b)

|   (a) |   (b) |
| --- | --- |

Figure A20 RMSE of RFG-TVIU for Scenes 3-1(a) and 3-2(b)

Appendix B

Table B1. Specific sensor metrics used in the experiment

| IMU parameters | Angular velocity | Acceleration | UWB parameters | Camera parameters | RTK parameters |
| --- | --- | --- | --- | --- | --- |
| Range | $\pm450^{\circ}s^{-1}$ | $\pm24g$ | The ranging error under LOS is 5 cm | see reference [9] | Positioning accuracy is 1-2cm |
| Zero bias stability | $2^{\circ}h^{-1}$ | $0.1mg$ |  |  |  |
| Zero bias repeatability | $\pm1^{\circ}s^{-1}$ | $\pm30mg$ |  |  |  |
| Random drift | $\pm0.014^{\circ}s^{-1}$ | $230\mu g$ |  |  |  |
